# Addressing infectious diseases in Africa by accelerating drug discovery through data science

**DOI:** 10.1038/s43856-025-01211-z

**Published:** 2025-11-25

**Authors:** Gemma Turon, Jason Hlozek, Miquel Duran-Frigola, Kelly Chibale, John G. Woodland

**Affiliations:** 1 Ersilia Open Source Initiative, Barcelona, Spain; 2 Department of Chemistry and Holistic Drug Discovery and Development (H3D) Centre, University of Cape Town, Cape Town, South Africa; 3 South African Medical Research Council Drug Discovery and Development Research Unit, Institute of Infectious Disease and Molecular Medicine, University of Cape Town, Cape Town, South Africa

**Keywords:** Infectious diseases, Drug discovery

## Abstract

Despite being rich in natural resources and scientific talent, Africa continues to bear a staggering infectious disease burden. Historically, health innovation on the continent has relied on international funding and has been constrained by limited infrastructure and the emigration of skilled professionals. Data science tools offer a promising alternative, typically requiring fewer costly resources than traditional empirical research, with the potential to empower African scientists to generate tangible and impactful health solutions for the continent. Rapid progress in data science is expected to transform infectious disease research; thus, it is encouraging that numerous African initiatives are already applying data science tools to tackling pressing unmet medical needs, particularly in drug discovery. These efforts include identifying novel therapeutic targets, predicting drug-like molecules and their synthesis, enhancing clinical trial success rates and preparing for future disease threats. This review examines the current landscape of data science in infectious disease drug discovery across Africa.

## Abstract (web summary)

Turon et al. review the data science and research tool in relation to the heavy burden from infectious diseases, compounded by limited infrastructure in Africa. Data science is emerging as a powerful tool for advancing drug discovery and improving health outcomes across the continent, with promising case studies highlighting its growing impact.

## Introduction

### Africa’s infectious disease burden

The WHO African Region (referred to hereafter as ‘Africa’) suffers from a disproportionately high burden of disease and is the only continent on which communicable diseases claim more lives than non-communicable diseases^1^. The five biggest infectious ‘killers’ in Africa are acute respiratory infections, diarrhoea, HIV/Aids, malaria, and tuberculosis, which together are responsible for 60% of Africa’s total infectious disease burden, leading to more than 2.4 million deaths per year. The impact of these numbers is compounded due to survivors of infectious disease in Africa being much more likely to suffer from debilitating complications, with the African continent further accounting for a disproportionately high number (52.5%) of global disability-adjusted life years originating from infectious diseases^1^. According to the United Nations, Africa’s population is projected to quadruple over the next century^2^. Temperatures across the continent are also expected to rise between 3 °C and 4 °C (5.5 °F and 7 °F) over the next century, potentially leading to more drought, conflict and biodiversity loss, and raising the possibility of future pandemics through increases in the habitable ranges of animal disease vectors or by increasing pathogen survival time^3,4^.

Consequently, reducing the burden of infectious diseases in Africa calls for a multipronged strategy that includes better sanitation, broader access to vaccines, and affordable treatment options. However, many of those diseases, such as mycetoma, leishmaniasis or pneumonia caused by drug-resistant *Klebsiella*, remain without effective or accessible treatments and, even when therapeutic options are available, the continent still faces significant challenges in drug distribution and primary healthcare, aggravated by the emergence of drug resistance^5^. Additionally, the rich genetic diversity of the continent is often associated with varying responses to medication, resulting in suboptimal treatments or unforeseen adverse reactions. This issue is exacerbated by the predominant use of cohorts of European ancestry in clinical research as well as the absence of African-centric preclinical screening models and tools^6^. Therefore, incorporating African data into drug discovery and development is essential for advancing a research agenda that reflects the continent’s unique needs and context^7,8^.

### Infectious diseases prevalent in Africa

As the area for greatest potential impact, African-led drug discovery research primarily focuses on infectious diseases with local relevance but without adequate or optimal treatment options. These diseases are caused by a variety of pathogens and fall into the categories of neglected tropical diseases (NTDs, or those that have largely been overlooked by the international community) and emerging diseases (those first appearing or re-appearing in a population after a long period of absence). Importantly, there are other diseases that do not fit into either of these categories but remain major health issues for the continent, such as malaria, tuberculosis and HIV.

Parasite-borne diseases are particularly widespread in Africa and are challenging to treat as their causative agents typically possess complex life cycles, have animal host reservoirs and transmission vectors, and evade the human immune response through invasion of human cells. Malaria, leishmaniasis and schistosomiasis are three relevant examples. Africa carries 94% of the >200 million worldwide cases of malaria each year, with an associated healthcare cost of more than US$12 billion annually^9^. While malaria research and development (R&D) has attracted more resources from various international entities, R&D for leishmaniasis and schistosomiasis is severely limited. Leishmaniasis, a sandfly-transmitted disease, is endemic to areas inhabited by over one billion people, yet treatment options remain limited. Meanwhile, at least 90% of people at risk of being infected by schistosomiasis, mainly due to lack of adequate sanitation and potable water, live in Africa.

Bacterial diseases represent another prominent sphere of infectious disease R&D as resistance to existing antibacterials becomes increasingly pervasive; consequently, new compounds and treatment regimes are urgently needed. A primary research focus on the continent is the development of improved treatments for tuberculosis (TB), the top infectious disease killer caused by a single pathogen, *Mycobacterium tuberculosis* (*Mtb*), and a concerning example of the appearance of antimicrobial-resistant strains. Existing treatment regimens have lengthy periods of up to 6–9 months for drug-sensitive TB and 18–24 months for drug-resistant TB, often with intolerable side effects. Salmonellosis is another devastating zoonotic infection caused by *Salmonella* bacteria and causing almost 50,000 deaths per year in Africa. Lastly, the *ESKAPE* pathogens represent a set of highly infectious and multidrug-resistant bacteria that have been prioritised by the WHO for urgent development of novel therapeutic agents before all existing treatment options fail. In 2019, the sub-Saharan Africa region harboured the highest burden of antimicrobial resistance-related deaths at 23.5 deaths per 100,000 inhabitants compared to other regions^10^.

### Challenges for drug discovery in Africa

Drug discovery is a notoriously risky, expensive, and time-consuming enterprise, contributing to the assumption that it is unsuitable or unrealistic in Africa. Indeed, after the high attrition rates of the discovery phase, the probability of successfully transitioning a compound from Phase I clinical trials to regulatory approval is only around 10%^11^. The total mean cost from discovery to regulatory approval has recently been estimated to be $1.6 billion^12^. As a result, therapeutic areas such as infectious diseases, which present their own scientific challenges for drug discovery^13,14^ and that do not yield the desired return on investment, often struggle to secure funding. Although Africa showcased its capabilities in disease surveillance, pathogen sequencing^15,16^ and clinical trials^17^ during the Covid-19 pandemic, continent-wide integrated drug discovery is the critical ‘missing link’ in the biomedical value chain.

To face the formidable challenges associated with the drug discovery enterprise, Africa must first strengthen its research and higher education capabilities. Across much of the continent, universities remain constrained by poorly-equipped laboratories, limited shipping services, unstable power supply and unreliable internet connectivity. Weak procurement chains and a lack of locally-produced supplies mean that it is more difficult and expensive to bring reagents and equipment into the continent^18^. The few available specialised instruments are often shared between institutions from multiple countries and are limited in capabilities, as it is common for them to have been donated as retired instruments from facilities in the developed world^19^. With some notable exceptions, the meagre infrastructure and support for research are dismaying for African PhD-holders^20^, with many who return home to Africa after a stint abroad facing challenges to be competitive in the global research landscape^21^. Others never return, contributing to the continent’s ‘brain drain’^22^, the emigration of skilled nationals resulting in a depletion of human resources in their countries of origin.

Despite ambitious training programmes around the continent, Africa still lacks a critical mass of skills in science and health innovation. The number of tertiary graduates in Africa is projected to rise from 103 million to 240 million by 2040, highlighting the urgent need to provide local job opportunities to the next generation of scientists^23^, replete with appropriate financial compensation. Alarmingly, scientific output from the continent represents less than 1% of the global share^24^ and the sub-Saharan Africa region invests only 0.5% of its gross domestic product, less than a third of the world’s average (1.8%)^25^. Consequently, drug discovery efforts rely on international funding agencies and depend on them to establish priorities and research goals, affecting local leadership^26^.

### The promise of data science for African drug discovery

In recent years, boosted by the outstanding progress of artificial intelligence (AI), data science has become an essential component of the biomedical research enterprise. Arguably, Africa is the region that may benefit the most from these advances, offering its citizens the tantalising possibility of technological ‘leapfrogging’ to close the gap with wealthier nations in the Global North. AI methodologies analyse existing data for patterns that can then be used for predictions and insight into novel data points, including the identification of potential drug leads prior to chemical synthesis and testing. This can be an enabler in resource-constrained settings where the cost of laboratory experiments is often prohibitive. Drug discovery—a field that is eminently interdisciplinary and relies on cumulative evidence to progress compounds to the clinic—has particularly benefited from AI methods^27,28^. With sustained funding, world-class infrastructure and abundant data, the field has been spearheaded by non-communicable disease research in the Global North—as demonstrated by a plethora of AI-developed drugs progressing to clinical trials^29^. Arguably, poorly-funded research fields such as those in infectious diseases, and NTDs in particular, have the potential of benefiting the most from these promising advances^30^.

However, this is dependent on African countries investing in and providing access to the three main ingredients that underpin AI—affordable and reliable power, digital infrastructure, and data. The remainder of this narrative review will highlight the major advances in the development of AI/ML tools in drug discovery projects on the continent, and discuss the recent advances in the field that will help bypass the slow adoption of these novel methods to advance the creation of an African research ecosystem powered by data science and AI/ML.

## Drug discovery initiatives in Africa

Drug discovery is a highly integrated endeavour that requires cooperation amongst multiple scientific disciplines across chemistry, biology, pharmacology, and so on. There is no formal academic training to become a drug discovery scientist, which instead comes from on-the-job training after specialisation in a relevant PhD programme. However, as the African innovative pharmaceutical industry is still in a nascent phase of development, there is limited capability to foster and grow a critical mass of skills. Consequently, African contributions to drug discovery are typically limited to small, focused studies within a particular scientific discipline in the context of a postgraduate degree.

One of the most notable achievements in African drug discovery comes from the Holistic Drug Discovery and Development (H3D) Centre at the University of Cape Town in South Africa. Working in collaboration with the Medicines for Malaria Venture (MMV), H3D spearheaded an international research effort that produced the first small-molecule drug candidate to be discovered and advanced into clinical development entirely within Africa. This candidate, known as MMV390048, moved into Phase II trials with African patients, marking progress not only in malaria treatment but also in the continent’s capacity for both fundamental and translational research. Importantly, MMV390048 represented the first *Plasmodium* kinase inhibitor to reach clinical testing—a significant milestone given that kinases have traditionally been targeted for cancer drug discovery rather than for malaria^31,32^. The achievements of the H3D Centre highlight that fostering a thriving ecosystem for health innovation depends on strong partnerships at both local and global levels; such partnerships bring together governments, universities, product development partners, philanthropic bodies and industry stakeholders^33^. To ensure lasting impact, these collaborations should be complemented by a human-focused strategy in which research institutions actively cultivate and strengthen scientific leadership capabilities.

In 2018, the African Academy of Sciences launched a collaboration through its Grand Challenges initiative together with the H3D Centre, MMV and the Gates Foundation. The joint initiative set out to strengthen the foundations of drug discovery across the continent, giving rise to the Grand Challenges Africa Drug Discovery programme. Central to this effort was the creation of capable research teams, supported not only by funding but also by exposure to industry expertise and mentorship. Sixteen projects were awarded grants^34^, with the H3D Centre providing continued guidance and capacity-strengthening support for participating groups. In 2023, the Gates Foundation and LifeArc jointly invested $7.2 million towards selected projects over 3–5 years as part of the Grand Challenges African Drug Discovery Accelerator (GC ADDA) network established with the H3D Foundation^35^. The GC ADDA grantees represent eight different African countries with two flagship projects addressing malaria and tuberculosis drug discovery. Funding has also been provided to support an African drug metabolism and pharmacokinetics research network and the assembly of an African-derived natural products library. A portion of the funding has gone to the H3D Foundation, established in 2019 to strengthen the ability to attract, develop and retain talented African researchers in innovative R&D, thereby supporting the GC ADDA network, with the H3D Centre serving as its technical partner.

A snapshot of some of the recent work contributing to African-flavoured drug discovery and development across the continent reveals advances from fields as diverse as basic pathogen biology (probing field isolates of malaria in Mali to better understand *P. malariae* intra-erythrocytic development and invasion^36^) to the use of *P. falciparum* field isolates to optimise the selection and combination of dose regimens for antimalarial treatment^37^. Critical efforts to better understand the safe and efficacious use of medicines in populations of African ancestry is being conducted as part of the work of the Zimbabwe-based African Institute for Biomedical Science and Technology^38^, while elucidation of genome sequences of local strains of pathogens, for example, for *S. aureus*, provides a valuable data resource for the development of new vaccines^39^.

Although initial efforts to build and strengthen capacity have been focused on individual African researchers, new centres of research have begun to emerge. In 2022, a collaborative drug discovery hub was launched in West Africa, coordinated by the University of Ghana together with the Noguchi Memorial Institute for Medical Research. Its starting priority is malaria drug discovery. Over the longer term, the hub seeks to expand its facilities and expertise to support end-to-end drug discovery, including compound screening, optimisation and characterisation, with the ultimate goal of delivering preclinical drug candidates^40^. A further emerging drug discovery centre based in Cameroon (Central Africa), the University of Buea Centre for Drug Discovery, is currently investigating putative antiviral compounds against HIV and SARS-CoV-2 from natural product sources^41^.

## Leveraging data science for drug discovery in Africa

Traditionally, drug discovery begins with an identified ‘hit’ compound that is found by phenotypic or target-based high-throughput screens of thousands or even millions of compounds. This is followed by costly and resource-intensive rounds of experiments and optimisation, including human clinical trials, until the drug reaches regulatory approval. Some of the ways in which AI and data science are proposed to accelerate the traditional drug discovery pipeline are listed in Table 1, with those of particular relevance to the African context highlighted.

**Table 1:** The major phases of the drug discovery and development value chain, with approximate timescales in the absence of AI or data science tools

Target identification and validation (1–2 years)	Hit identification and lead optimisation (2–5 years)	Preclinical testing (1–2 years)	Clinical trials (6–10 years)	Regulatory approval and post-marketing surveillance (1–3 years)
• **Examine genomic datasets to uncover factors contributing to diseases prevalent in Africa, to identify novel therapeutic targets and personalised treatments tailored to the genetic diversity of African populations**. • Integrate analyses of biological interaction networks and chemical information to identify key genes or proteins involved in disease pathways, supporting both target discovery and validation. • Apply predictive models to estimate the ‘druggability’ of a target (i.e. the likelihood of it being successfully modulated by a small molecule) to streamline drug discovery efforts.	• **Virtual screening of large compound libraries to identify molecules with promising biological activity, particularly in the case of natural products for which scaffold-hopping strategies can support synthetic accessibility**. • **Explore opportunities for repurposing and repositioning existing drugs for diseases that disproportionately affect African populations**. • **Devise synthetic routes tailored to available reagents in order to reduce costs and avoid delays linked to reagent procurement**. • **Employ predictive models for absorption, distribution, metabolism, excretion and toxicity (AMDET) to prioritise molecules with favourable profiles**. **•** Generate new chemical structures that are computationally optimised for desired drug-like properties to accelerate the transition from a hit to a lead compound. **•** Predict the binding affinity between potential drugs and their target proteins to support optimisation efforts.	• **Anticipate potential toxicities early in the development process to reduce the need for costly animal studies**. **•** Identify biomarkers linked to treatment outcomes or disease progression, aiding preclinical testing and patient stratification.	• **Predict patient responses to treatments in order to enable adaptive trial designs and optimal dosing regimens**. **•** Assess patient datasets to match individuals to clinical trials according to genetic profiles, disease characteristics and other factors, leading to more efficient recruitment.	• **Track real-world health data to detect adverse reactions or drug interactions, strengthening post-approval monitoring and ensuring patient safety**.

Examples of ways in which AI and data science tools are expected to accelerate this pipeline are listed, with those that are particularly relevant to the African context **highlighted in bold**.

A recent Wellcome Trust report on the potential of AI in drug discovery identified three major use-cases of data science in small-molecule drug discovery: (i) identifying and validating novel drug targets; (ii) small molecule design and refinement; and (iii) the evaluation of safety profiles of therapeutics^42^. In the African context, the potential is augmented by the fact that (i) known therapeutic targets are critically lacking for (neglected) pathogens, (ii) the intensive rounds of experimentation and throughput necessary to design new medicines are unfeasible locally, and (iii) African populations are not adequately represented in clinical trials, increasing treatment risks. Of these three points, the preclinical ones—namely, target identification and molecular design—are by far the most thoroughly explored.

A distinction ought to be made between data science for antimicrobial drug discovery globally, and data science efforts for which the scientific leadership resides in Africa. Globally, AI has already shown promise in prioritising broad-spectrum antimicrobials^43^, as well as compounds against *Acinetobacter baumannii*^44^, providing genuinely new chemical entities for further exploration. Alongside these advancements, generative AI tools capable of systematically exploring the antimicrobial chemical space are emerging^45^. The field is expected to continue benefiting from AI applications, including large language models (e.g. GPT), image analysis for phenotypic screening data, and comprehensive processing of ‘omics’ data. This approach is likely to draw from the progress made in other well-established areas, like anticancer drug discovery, in which various data types—including transcriptomics profiles and genetic screenings—are integrated to forecast drug activity seamlessly^46^. Additionally, we anticipate increased development of host-directed therapies, which either block pathogen infection or stimulate the human immune system.

Unfortunately, although the ultimate goal of much of this research is to meet the healthcare needs of Africa, the majority, if not all, of it has originated and been developed outside the continent. It may be unrealistic to expect that Africa will generate all the necessary data to ‘train’ AI models for drug discovery in a standalone fashion. Creating large datasets, which are crucial for the success of AI tools, is often prohibitively expensive, even for a single centre in the Global North. Indeed, initiatives like the Tuberculosis Drug Accelerator^47^, the Malaria Drug Accelerator^48^, MMV and CO-ADD^49^ rely on data gathered from multiple centres around the world. Thus, in the context of decolonising research, drug discovery presents unique challenges compared to other fields such as epidemiology and vector control, in which data production occurs on-site, and local strategies are necessary to leverage these data effectively. In drug discovery, and especially preclinical drug discovery, bioactivity screening data is ideally produced with high throughput in large facilities worldwide; the challenge is to make this worldwide data actionable in Africa, ideally in the form of AI tools deployed on-site or accessible via free or affordable online inference services.

In our experience, adoption of AI can be challenging and particularly slow in Africa. Often, no computational skill sets exist on premises to support the implementation of AI tools, which tend to demand advanced technical expertise and frequently require fine-tuning to a particular problem of interest. Additionally, AI tools are heavily reliant on datasets of sufficient volume and quality, which are typically not made accessible to the broader scientific community, and user-friendly proprietary implementations of AI often require prohibitively expensive licenses that prevent access in resource-constrained institutions. To further discuss these matters, we now expand on the various factors that contribute to the successful implementation of AI for global health and consider the current state of the field in Africa.

A recent bibliometric analysis shows that between 2013 and 2022, of the top ten countries publishing about ‘AI in Africa’, only two, South Africa and Nigeria, are actually located on the African continent^50^. This demonstrates that AI development in general is still in its infancy in Africa. Nevertheless, in the last few years, the combination of academic, non-profit and small start-ups has rendered the first promising results, with South Africa emerging as a leading hub for the application of AI to drug discovery. We recently published the first end-to-end implementation of a virtual screening cascade for malaria and tuberculosis at the H3D Centre in South Africa in collaboration with Ersilia^51^. Similarly, scientists at the University of Pretoria in South Africa are also developing AI/ML models to forecast the antimalarial potential of new drug candidates^52,53^. With the support of Collaborative Drug Discovery Inc., a US-based pharmaceutical software firm with a strong philanthropic ethos, other research groups across Africa have started establishing AI/ML virtual screening pipelines for tackling tuberculosis^54^. Aside from the three major disease areas (malaria, tuberculosis and HIV), modest developments towards adoption of data science and AI/ML for NTDs are also happening on the continent; a recent perspective describes the benefits, limitations, and pitfalls of AI/ML tools for antiviral drug discovery^41^, and AI could also accelerate the identification of therapeutic options for Ebola virus disease^55^.

Data science presents an avenue to circumvent the inherent challenges of infectious disease research, compounded by a lack of data, difficult model systems, and poor local scientific infrastructure in the African context. Moreover, emerging AI/ML methods have the potential to reduce overall development costs in low- and middle-income countries, paving the way for locally-d-eveloped, affordable drugs from within these regions.

### Data availability

Effective application of AI/ML methods to drug discovery is highly dependent on sufficient and high-quality data, including the outcome of phenotypic assays, receptor-ligand interactions, toxicity of compounds and, whenever available, clinical data. Most laboratories across Africa do not possess the funding and infrastructure to perform high-throughput experimental assays and must resort to publicly available data as a starting point for AI/ML model training; for example, available from ChEMBL^56^, PubChem^57^, DrugBank^58^ and BindingDB^59^. This presents several challenges, starting with data curation from multiple sources, and requiring more careful interpretation of the models, ensuring the results obtained from an AI/ML model trained with external data can be confidently applied to the chemical space and experimental conditions of interest. Indeed, recent advances in AI/ML can leverage the scarce data existing for poorly-studied diseases to enhance research in low-resourced settings. Those include transfer learning techniques, in which neural networks are pre-trained on larger corpuses of data and then fine-tuned to the specific task at hand^60^, and few-shot and zero-shot learning techniques devised to learn from very few labelled data points^61,62^. In addition, the development of large language models (LLMs; including LLAMA, GPT or BLOOM) opens the door to using unstructured text as inputs for AI/ML modelling^63,64^. These novel methods hold promising potential to speed up research in the low-data scenario for infectious diseases.

### AI-based target identification

Pathogen biology for most of the causative agents of NTDs is largely unexplored, partially due to the complexity of in vitro culture (for example, for *Cryptosporidium* species^65^) or the lack of adequate animal models, even for better-studied diseases like malaria^66^. Without proper parasitology studies, target identification remains a challenge, and most drug discovery pipelines necessarily need to start by phenotypic, rather than target-based, assays. Advances in AI-driven tools for predicting protein structure, such as AlphaFold^67^ and ESMFold^68,69^, are bringing the field closer to performing molecular docking experiments on proteins for which crystallographic data are lacking. This approach has already elucidated a resistance mechanism in *P. falciparum* linked to mutations in the *Pf*ATP4 ion channel^70^, uncovered new therapeutic targets in *T. cruzi* (the parasite responsible for Chagas disease)^71^, and supported investigations into a range of viral proteins^72^. Nevertheless, caution should be exercised when using those novel tools, as docking-based approaches demonstrate that AlphaFold predicted proteins might not be accurate enough^73^. Current efforts by the spin-off from the developers of AlphaFold, Isomorphic Labs, are focused on improving model performance^74^.

Finally, advances building on AlphaFold are accelerating the mapping of host-pathogen interactions by revealing molecular complexes^75^ and, when combined with network biology approaches, they offer powerful tools for identifying critical targets in both the host and the pathogen^76,77^. To name an example, an *Mtb*-human protein-protein interaction involving 34 proteins secreted by *Mtb* revealed a switch between the host’s antiviral and antibacterial immune responses, which can be further exploited therapeutically^78^.

### Data science infrastructure

The development of biomedical research data science centres in Africa might seem a challenging endeavour, given that many countries still face unreliable electricity and internet supplies^79^, which can disrupt data collection, storage and processing. These disruptions not only compromise the integrity of health data but also limit the deployment of advanced health technologies. Ensuring widespread access to cost-effective and dependable electricity and access to the internet, particularly in rural and under-resourced areas, is therefore essential to unlocking the full potential of data science for health systems strengthening in Africa.

Fortunately, recent technology developments are providing several avenues to bridge this gap. On one hand, the rise of local AI agents, i.e. AI-based models that run entirely in the user’s hardware, is bringing LLMs to areas with poor internet connectivity and low computational infrastructure^80^. For example, the Mozilla-backed initiative Llamafile already provides locally executable files for many LLMs. On the other hand, the growth of cloud computing providers allows researchers to leverage scalable, graphics processing unit-accelerated computing systems from their local institutions^81^ and, by placing their computational pipelines in remote servers, bypasses the danger of electricity and internet cuts, and reduces the need for expensive solar or generator back-up systems. In sum, technological advances provide a great avenue for the nascent data science landscape in Africa, yet these advances must be coupled with the support of international initiatives such as the recent NIH Harnessing Data Science for Health Discovery and Innovation in Africa^82^, as well as the investment of local governments^83^.

### Open-source drug discovery

Finally, the open-source paradigm, i.e. releasing data, code and results in real-time so that researchers can contribute to the project as it develops, and not only upon publication, is uniquely well-suited to facilitate the development of data science for infectious disease research in Africa. Open-source drug discovery harnesses collective contributions—by sharing data, software and methodologies—to find patent-free drugs. This approach has been pioneered by the Open Source Malaria consortium, which is actively working on several series of potential antimalarial hits^84^, and has expanded to other disease areas, including antibiotic-resistant bacteria (OSA) and the fungal infection mycetoma (MycetOS). Those initiatives provide engagement opportunities via computational challenges, including the DREAM Challenges, and result in a plethora of novel open AI/ML tools for malaria and other diseases^85,86^. When paired with open-source platforms that make these tools accessible to non-specialist researchers—such as the Ersilia Model Hub^87^—this approach creates opportunities for African scientists to form collaborative networks of data scientists, software developers, medicinal chemists and biologists, all guided by the principle of open source and open science. The recently published Covid Moonshot project demonstrates how a crowdsourcing approach to drug discovery can accelerate the finding of new drugs at low cost^88^.

## Opportunities in an African context

**Fig. 1:**
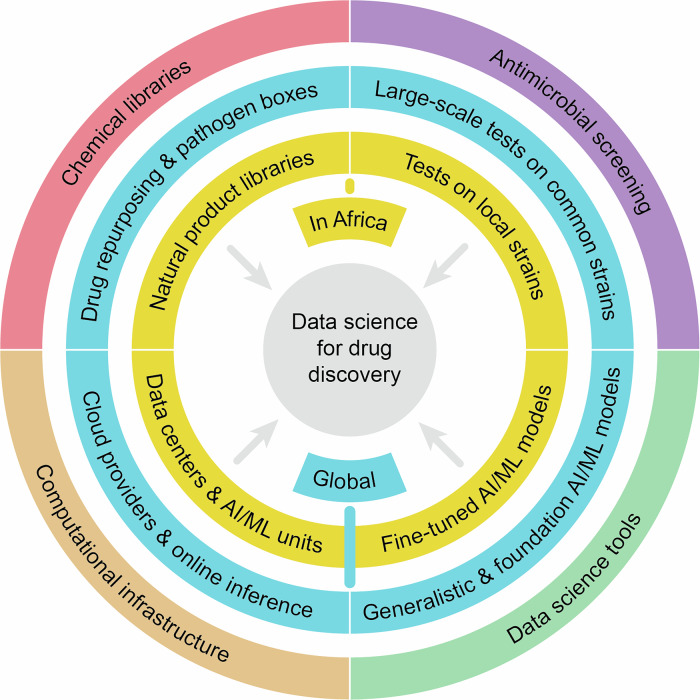
An illustration capturing the various resources and factors contributing to the successful implementation of data science tools for African and global health in the context of infectious disease drug discovery.

The absence of a local innovative pharmaceutical industry on the continent became apparent during the Covid-19 pandemic, as African countries were mostly reliant on treatment and vaccine development from outside the continent^89^. As demonstrated by the aforementioned Covid Moonshot project and the Canadian-led prostate cancer treatment development^90^, computational tools can provide an advantage to resource-limited areas of research, creating a unique opportunity to expedite Africa’s drug discovery research agenda. Particularly, two avenues for leveraging those methods in Africa stand out in the context of infectious disease (Fig. 1).

On one hand, drug repurposing, in which existing drugs are investigated for use in a different therapeutic area, is an attractive strategy that aims to reduce the time and costs of pharmaceutical research by leveraging existing data to advance quickly through the early drug discovery stages. Since drug repurposing depends on extensive data from existing drugs and diseases, data science tools hold promise in allowing researchers to, for example, automate the identification of active ligands and prioritise multi-target scores for promising molecules. While this could be a boon for polypharmacological investigations, issues of selectivity and toxicity still need to be considered.

Secondly, natural products remain an untapped source of novel compounds, particularly in Africa, which boasts a vast array of plants and animals with potential medicinal properties^91–93^. Work in this field has led to several natural product databases, such as NANPDB and SANCDB, that can be used as an initial data source for virtual screening and prioritisation for further investigation^94,95^. Natural products, once identified, are typically complex to access synthetically and are often computationally processed through various scaffold-hopping approaches to identify a more drug-like pharmacophore for further exploration^96^. Finally, traditional and indigenous knowledge remains a source of undisclosed potential for finding new therapeutic options for endemic diseases in Africa. Almost 5000 plants are used in African traditional medicine^97^, and the first examples of successful collaboration between scientists and traditional healers are emerging^98^. Data science and AI/ML techniques can also be employed to systematically collect, organise and analyse the wealth of traditional medicine and indigenous knowledge to identify promising targets or compounds based on traditional uses.

While the rich genomic diversity of the continent’s population may appear to be a complicating factor in treating disease in Africa, it also represents an opportunity to deliver highly optimised therapies. To advance this goal of precision medicine, the Human Heredity and Health in Africa consortium has invested in developing biorepositories for African-specific genome data while building a pan-African bioinformatics network, H3ABioNet, to advance computational biology research on the continent^99^. Growing the pharmacogenetics knowledge base to better understand the interplay between African genetic diversity and treatment outcomes relies on data science approaches to identify prevalent variants of pharmacogenes in African populations relevant to drug metabolism in order to optimise drug dosage for African patients, as exemplified by Project Africa GRADIENT^100^. While little data are, in principle, required for this approach, it is critical that infrastructure, trained staff and secure databases are developed and maintained.

Once prospective treatments advance to the clinical stage of research, it is important for compounds to be tested in representative populations to ensure that safety and effectiveness can be accurately assessed before regulatory approval. However, like the challenges faced in early drug discovery research, Africa lacks the critical mass of infrastructure, skilled clinical research practitioners and funding to facilitate this research at scale. It comes as no surprise, therefore, that despite being home to 17% of the global population, only 3% of clinical trials take place on African soil^101^. To bridge this gap, the African Union established the African Medicines Agency in 2021 to advance the goal of a standardised legal and ethical framework for robust regulatory review of medical research in Africa^102^. As AI methodologies are broadly applicable across datasets, data science tools offer much promise at multiple points in the clinical research pipeline by assisting with trial design, participant enrolment, patient monitoring and analysis of clinical end-points^103^.

## Outlook

While infectious diseases remain a significant burden on health in Africa, the emergence of data science tools has opened new avenues for accelerating drug discovery in the region. In this review, we have outlined the crucial role of data science tools and their diverse applications in infectious disease drug discovery on the continent. ML algorithms, data mining and computational modelling offer powerful approaches for analysing complex biological data, identifying potential drug targets, and expediting drug development. Integrating genomics, proteomics and epidemiological data will allow for a holistic understanding of pathogens and disease progression to facilitate the prioritisation of drug candidates tailored to Africa’s unique epidemiological landscape. Various drug discovery initiatives are flourishing on the continent, and these should be accompanied by data science tools and pipelines adapted to their needs.

Leveraging these data science assets will require collaborative efforts amongst researchers, clinicians, public health experts and data scientists. Initiatives that foster interdisciplinarity and data sharing will enhance the accessibility and usability of these tools, leading to more effective interventions and improved health outcomes. While barriers to the adoption of data science tools in the African context, such as infrastructural constraints and the need for capacity strengthening in computational and data science skills, should not be neglected, overcoming these challenges will require concerted efforts from funding agencies, academic institutions and policymakers to invest in infrastructure development, data governance frameworks and educational programmes tailored to the needs of the African community. Importantly, risks and challenges such as bias, safety, model explainability and governance should not be overlooked.

Data science tools have the potential to transform infectious disease drug discovery in Africa; indeed, they are already doing so. In this review, we have highlighted a few representative examples of drug discovery initiatives primarily driven by African researchers, which, when compounded and complemented by valuable global efforts such as those of the Global Health Innovation Technology Fund and the Tokyo International Conference on African Development, set the stage for a strong and data-rich drug discovery community in the region. By harnessing the power of data science tools, the efficiency and effectiveness of drug discovery efforts can be augmented to improve public health outcomes across the continent. It needs to be emphasised that the provision of access to affordable and reliable power, digital infrastructure and data—complemented by skilled human resources—are the basic ingredients needed for data science tools to help leapfrog progress and avoid widening the inequalities between Africa and the rest of the world.

### Reporting summary

Further information on research design is available in the Nature Portfolio Reporting Summary linked to this article.

## Supplementary information

Reporting Summary

## Data Availability

The data that support the findings of this review were derived from resources available in the public domain.
